# Optic Perineuritis in Neuromyelitis Optica Spectrum Disorder

**DOI:** 10.7759/cureus.4834

**Published:** 2019-06-05

**Authors:** Nur Afiah Kamaluddin, Evelyn Tai, Wan-Hazabbah Wan Hitam, Mohtar Ibrahim, Ahmad Hadif Zaidin Samsudin

**Affiliations:** 1 Ophthalmology, School of Medical Sciences, Universiti Sains Malaysia, Kubang Kerian, MYS; 2 Radiology, School of Medical Sciences, Universiti Sains Malaysia, Kubang Kerian, MYS

**Keywords:** optic perineuritis, optic neuritis, nmosd

## Abstract

Optic perineuritis (OPN) involvement in demyelinating disease is rarely encountered. To our knowledge, this is the first reported case of bilateral OPN associated with neuromyelitis optica spectrum disorder (NMOSD). We present a case of a healthy young gentleman who presented with OPN, initially presumed to have a young stroke but later diagnosed to be NMOSD. Early neuroimaging is essential to help distinguish optic neuritis (ON), and prolonged treatment of systemic immunosuppression is the mainstay of treatment.

## Introduction

Neuromyelitis optica (NMO) is an autoimmune inflammatory disorder of the central nervous system which mainly affects the spinal cord and optic nerve. NMO spectrum disorder (NMOSD) is a subset form of NMO which did not meet the 2006 revision of the Wingerchuk criteria for NMO. Among Asians, East Asian origin populations (Chinese and Japanese) were known to have higher NMOSD prevalence than other Asian ethnic groups. Optic nerve involvement is usually in the form of optic neuritis (ON). We report a case of NMOSD presenting with bilateral optic perineuritis (OPN).

## Case presentation

A 35-year-old Malay male with no past medical illness presented with sudden onset of right facial asymmetry and slurred speech, followed by progressive right-sided body weakness over four days. The weakness started at the right upper limb and was followed by the right lower limb. He was initially treated with statin and aspirin for a presumed diagnosis of young stroke. One month later, he started to have bilateral, painless blurring of vision, noted upon waking from sleep. It was associated with headache and episodes of vomiting. He had a history of retrobulbar ON four years ago which fully resolved with a course of steroids. He denied any family history of neurological disorders.

At presentation, the visual acuity was hand movement in the right eye and no perception of light in the left eye. The anterior segment was unremarkable. The posterior segment revealed bilateral temporal optic disc pallor with well-defined disc margin. The relative afferent pupillary defect was positive in the left eye. Other optic nerve function tests were not performed due to poor visual acuity. Neurologically, he had global aphasia, gaze preference to the left side, a right facial upper motor neuron lesion, right hemiplegia, and a positive right Babinski reflex.

**Figure 1:**
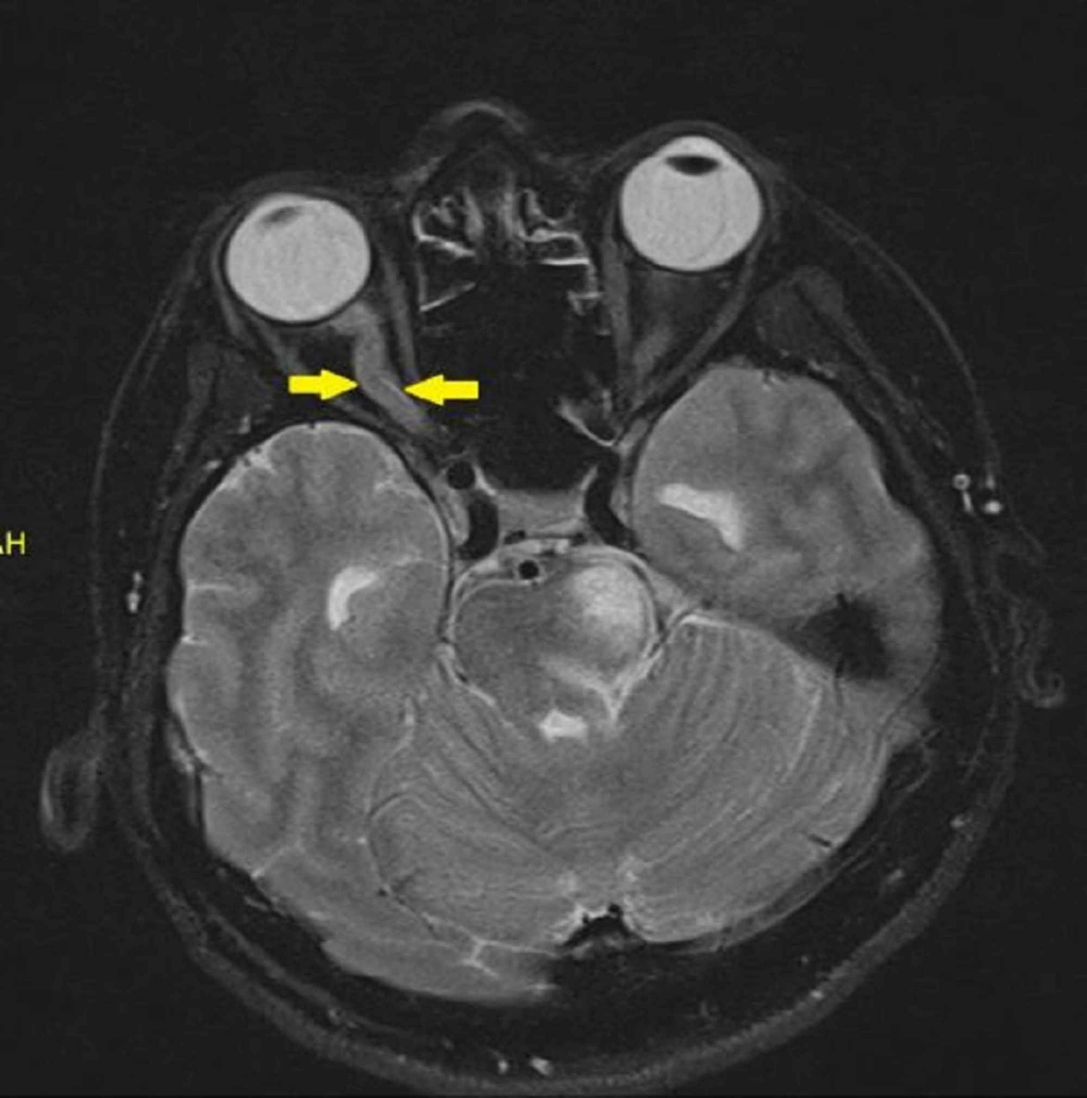
Magnetic resonance imaging (MRI) showed kinked right optic nerve with thickened optic nerve sheath

**Figure 2:**
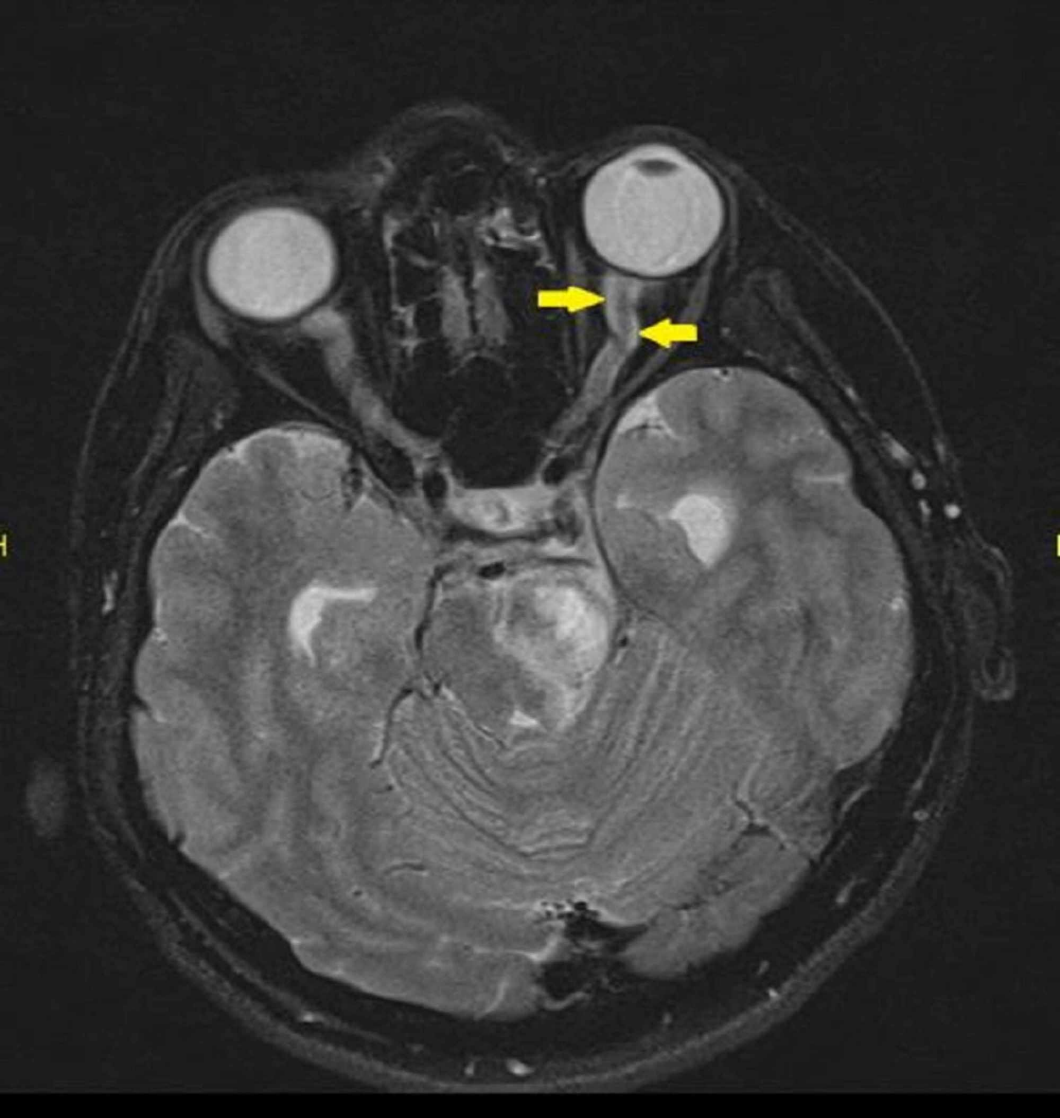
Magnetic resonance imaging (MRI) showed kinked left optic nerve with thickened optic nerve sheath

**Figure 3:**
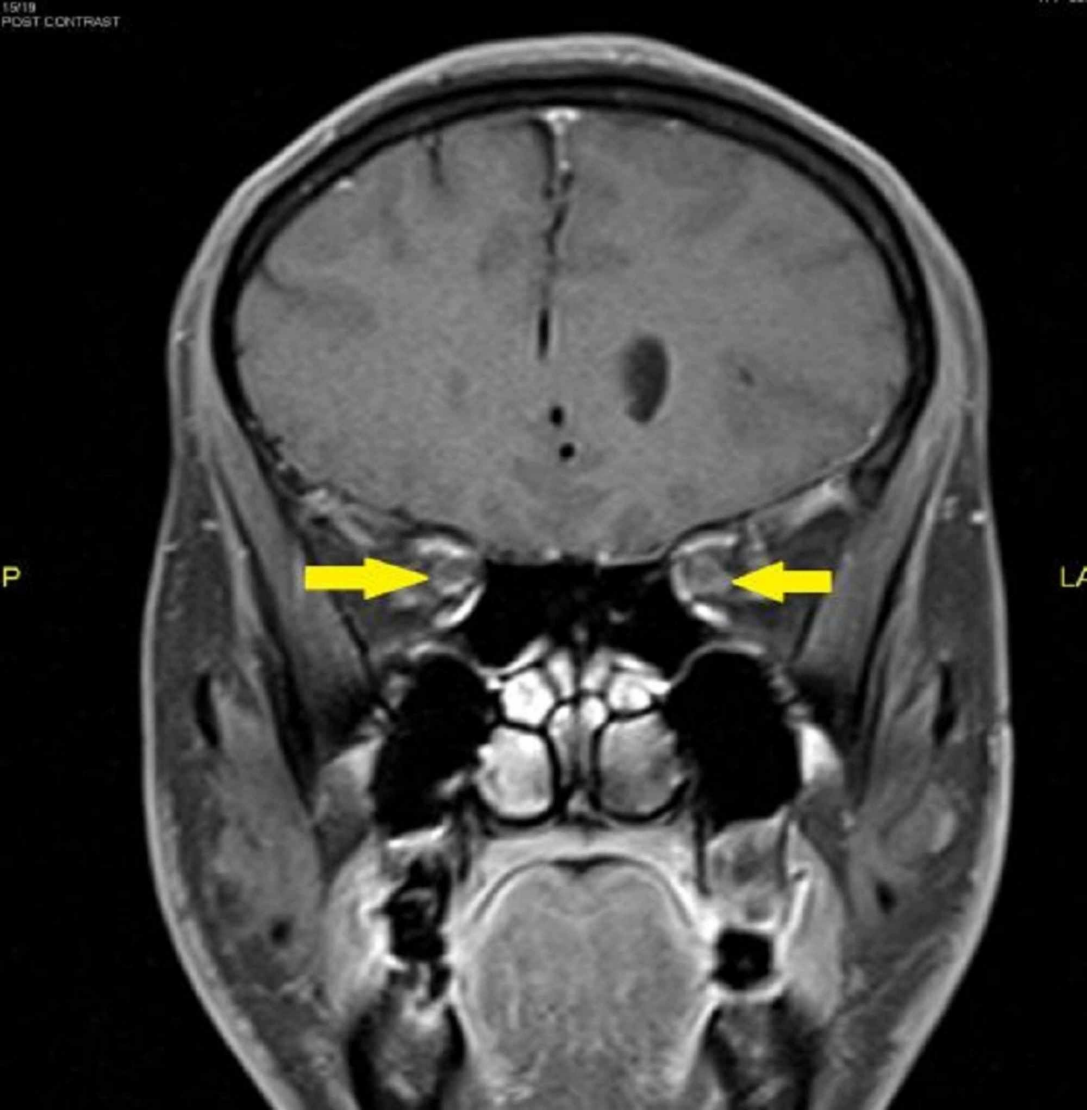
On coronal post gadolinium image, these optic nerve sheaths showed enhancement

Magnetic resonance imaging (MRI) of the orbit showed kinking of both optic nerves. The optic nerve sheaths were thickened and enhanced post contrast in keeping with OPN (Figures 1-3).

**Figure 4:**
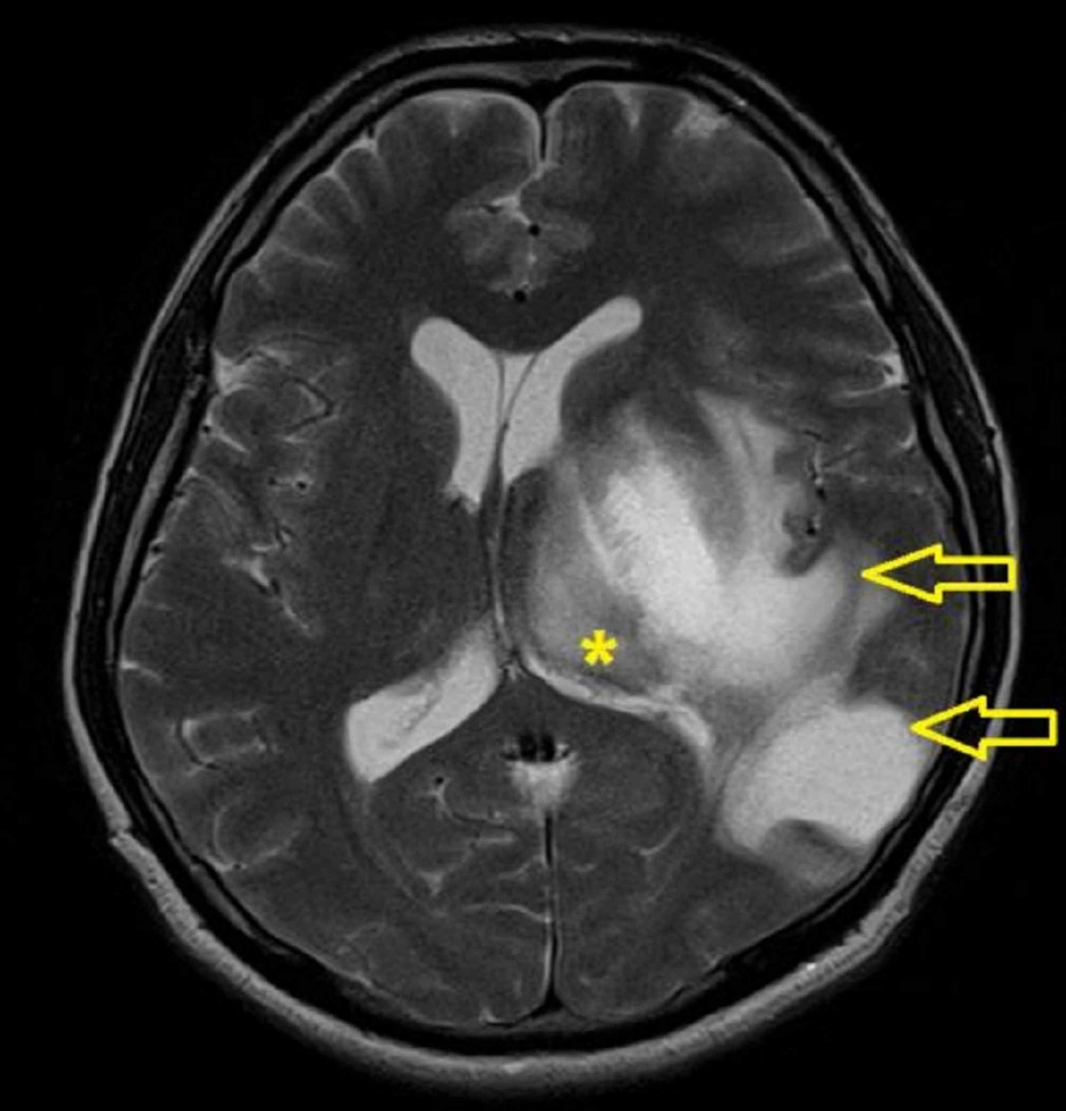
Magnetic resonance imaging (MRI) of the brain showed increased intensity within the left thalamus (*) and the left temporoparietal region (open arrow)

His brain MRI with diffusion-weighted imaging (DWI) sequence done showed lesions within the left thalamus, midbrain, pons, cerebral peduncle, and left temporoparietal occipital region. The lesions were hypointense on T1, hyperintense on T2WI and not enhanced post contrast (Figure 4). The brain lesions showed minimal restriction at the periphery region.

All relevant investigations to rule out different causes of cerebrovascular accident were negative, including thrombophilia screening and viral screening. Electrocardiogram (ECG) was normal. Other haematologic parameters such as full blood count, renal and liver profile, rheumatoid factors, antinuclear antibody, fasting lipid and blood sugar, and thyroid function test were normal. The erythrocyte sedimentary rate (ESR) was normal at 12mm/hour. The neuromyelitis optica antibody was negative. The progressive nature of the disease, involvement of the optic nerve, low ESR, brain lesions showed in the MRI as mentioned, and exclusions of other diagnosis were suggestive of NMOSD.

Intravenous methylprednisolone 1000 mg daily for five consecutive days was started, followed by oral prednisolone of 1 mg/kg/day, tapered later to a maintenance dose of 10 mg daily. Concurrently, oral azathioprine 50 mg daily was started after one month and increased later to a maintenance dose of 100 mg daily. Both medications were continued for a one-year duration. He was also prescribed with oral mecobalamin. Visual acuity of both eyes improved to 6/6, and at one year, the vision was maintained with only minimal residual optic nerve function defects in the left eye. He had no side effects of the medication, and his systemic neurological condition also improved.

## Discussion

OPN is an orbital inflammatory disease primarily affecting the optic nerve sheath and the surrounding tissue [1]. It is a great mimicker of typical ON. OPN has been reported mostly as idiopathic and isolated cases [1] although associations with other diseases such as Wegener granulomatosis [2], giant cell arteritis [3], Crohn’s disease [4], Behcet disease [5], leukemia [6] and acute retinal necrosis [7] have been reported. To the best of our knowledge, there are limited reports of OPN in patients with NMO or NMOSD.

The initial presentation of OPN can be indistinguishable from ON. Both can present with signs of optic nerve dysfunction such as reduced color perception, and swollen or normal-appearing optic nerve head [8]. However, in OPN, patients may have paracentral or arcuate scotomas rather than central scotoma in ON. Orbital signs such as diplopia, ptosis, ophthalmoparesis, and chemosis may also be associated [9]. Our patient presented with rapid, painless bilateral loss of vision together with right hemiparesis, and facial asymmetry. This is an atypical presentation for ON, warranting urgent neuroimaging [8].

As the clinical features of ON and OPN may overlap, the diagnosis is classically dependent on radiological imaging. MRI findings of OPN will show the characteristic of optic nerve sheath enhancement in the form of a ‘doughnut sign’ on coronal scans and ‘tram track’ sign on axial scans [9]. Besides, some may show enhancement of the optic nerve substance, which is due to the inflammation of the intraneural pial septa together with the nerve sheath. Some cases also may show additional streaky enhancement of the orbital fat with or without enhancement of the extraocular muscle and the sclera [1]. It has reported that the common orbital finding in a patient with NMOSD would be ON. In contrast with the OPN, MRI will show a nonspecific optic nerve sheath thickening, T2-weighted hyperintensities of the optic nerve, and gadolinium enhancement [10]. Diffusion‐weighted imaging (DWI) which was previously used for detecting acute stroke has increased its diagnostic role, particularly in the head and neck lesion. Its short acquisition times makes it easily incorporated into head and neck MRI protocols. In orbital imaging, it has a role in differentiating inflammatory from malignant causes [11]. Restricted diffusion has been reported in a patient with acute ON [12]. However, DWI images can have meager signal-to-noise ration due to the geometrical shape of the structures in the head as well as the presence of adjacent thick bones. It also can have strong susceptibility artifacts from the metallic surgical implant and air-tissue boundaries [11]. However, we were unable to interpret the diffusion images within the orbital region in this patient. Thus, this case highlights the importance of early neuroimaging in patients with apparent ON. Evidence of neurological impairment should also prompt consideration of NMOSD. A proper index of suspicion is required as the diagnosis has multiple systemic and prognostic implications.

Systemic corticosteroids are the traditional first-line therapy for acute phases of OPN as well as NMOSD. The goal of the treatment is to arrest the inflammatory process and to treat the underlying causes. In both cases of ON and OPN, intravenous methylprednisolone (1 g/day) for 3-5 consecutive days can be given in acute attacks, followed by oral therapy. In comparison with typical ON, prolonged steroid treatment for OPN is essential in preventing irreversible vision loss and recurrent attacks [13]. In NMOSD, recent consensus indicates that subsequently, oral prednisolone can be prescribed together with azathioprine (AZA) or mycophenolate mofetil (MMF) within the first months of the attack. Oral prednisone at 1 mg/kg/day dose is necessary for the initial period and can be slowly tapered when AZA (2-3 mg/kg body weight per day) reaches its full effectiveness after 3-6 months [14]. Further preventative immunosuppressive therapy includes rituximab, methotrexate, and intravenous immunoglobulin. These can be options in patients that do not respond to first-line therapy or suffer from side effects of other treatment.

## Conclusions

OPN in NMOSD is rarely encountered and may be associated with neurological impairment. Neuroimaging is essential in such cases. Prolonged systemic immunosuppression is the cornerstone of therapy. Prompt diagnosis and treatment increase the excellence of the prognosis for vision outcome like in this patient.
